# Dental Hints for Country Medical Practitioners

**Published:** 1879-12

**Authors:** G. Newkirk

**Affiliations:** Wenona, Ill.


### ARTICLE VI.

### DENTAL HINTS FOR COUNTRY MEDICAL PRACTITIONERS. By G. NEWKIRK, M.D., Wenona, Ill.

#### PART I.

It is probable that fully one-half your so-called "country readers" are located in towns or in the country where they are more or less remote from a resident dentist. A town may be large enough, in connection with the country tributary to it, for the support of one or two physicians, but not desirable as a location for a dentist; or if there be one, he may be grossly ignorant and incompetent. It is for physicians so located, and for the good of their patrons, that the following hints have been jotted down.

The country physician cannot avoid the necessity of paying some attention to carious teeth, much as he may desire to be rid of any responsibility connected with them. People *will* neglect proper attention to their teeth till the imperious mandate of pain compels them to seek relief from the dentist, if one be at hand, otherwise the nearest physician. They are generally ignorant regarding the nature of their dental organs, and usually greatly underrate their value; and it is to be lamented that physicians are often in this respect nearly as ignorant as their patients. With the city practitioner this ignorance matters not so much, for he can thrust off all cases upon the resident dentist; in fact he seldom sees them, thus avoiding nearly or quite all the responsibility which his country brethren are compelled to accept.

No reputable physician, in general practice at the present day, is disposed to assume any of the work properly belonging to a dentist, where there is one near at hand, but if there be none, he has no alternative but to do the best he can, both for the immediate relief and the permanent interest of his patrons. Here being a responsibility he cannot avoid assuming, what is his duty in the premises?

Plainly, he ought to place himself in the possession of such information, appliances and tools as are necessary in order to promote the best interests of his patients. He cannot, of course, assume to do the general work of a dentist, but he should do enough in that direction to put many teeth, such as are and have been commonly sacrificed, on the road to a competent dentist and salvation. He ought to be able to determine whether or not a tooth would be, in proper hands, probably salvable. He ought to be prepared, through knowledge and means, to give temporary relief to many aching or sensitive teeth, and temporary protection also, until some one specially skilled may be reached, who can make the relief and protection permanent. He ought to make himself thoroughly conversant with the special and relative anatomy of the jaws and teeth, and their nervous relations with other parts of the head and face. He ought to know the usual time and order of the eruption of both the temporary and permanent sets, the special form and position of each, both before and after eruption; this in order that he may be accurate in diagnosis and operative procedure, and least of all liable to mistake between a temporary and permanent tooth; that he may know how long the temporary tooth should be conserved, and when it may with advantage be removed. He should be sufficiently familiar with the special structure of each tooth and proper methods of diagnosis to be able to determine, upon examination, whether in a given case pain or uneasiness is caused by sensitiveness of dentine, a nearly or quite exposed pulp or inflammatory action in the alveolar socket (periodontitis). Without this knowledge, he may, on the one hand, sacrifice perfectly salvable teeth; or, on the other, attempt an impossible salvation, to the great discomfort of his patient, and humiliating annoyance of himself. The usual course of ignorance, however, is to commit destruction; the extraction of the tooth, however serious the loss to the patient, is felt to be an ultimatum, and on the principle that "dead men tell no tales," the ghastly heroic remedy is the one oftenest administered. But it is not a remedy to be always willingly administered by a man with a conscience.

But inasmuch as many of the teeth that are brought to us must of necessity be extracted, we ought to know so well the special forms and insertions of teeth, and the special forms of instruments to correspond, that we may always make a correct application of the one to the other, where it is our duty to use this means of relief.

For information, clear and exact upon this subject, every country physician should place himself in possession of the little book, "Robertson on Extracting Teeth," a treatise which is much more in fact than the name would suggest.

Now, what ought he to have in the way of tools and appliances?

As his operative procedures will be largely in the way of extractions, he ought to have a well-selected stock of forceps. (If he has a turnkey, let him present it to his butcher.) It is not at all necessary that he have a great number, but from the multitude of forms, it is essential that he shall know how to select the few he really needs. Many of the instruments placed upon the market are of very inferior quality, in both form and finish; many are applicable only in rare cases, and the purchaser must know how to *reject*, in order to *select*. And here let me say that whatever may be saved by the purchase of the cheaper instruments, will be but a trifling consideration for the difference in quality and the annoyances that will certainly be experienced in their use. It will be far better to get along for a time with half a set of first-class instruments, than to be in possession of a full array of those of inferior grade, each one of which shall be as "Confidence in an unfaithful man in time of trouble, is like a broken tooth and a foot out of joint."—Prov., 25 : 19.

It will be found also, that for cleanliness and freedom from rust, money can hardly be better spent than for the extra cost of nickel-plating. This is especially true as to forceps, and the extra cost will be about fifty cents each.

Let me remark first in this connection, that by far the greater number of teeth you will feel compelled to extract, as many of you know by experience, will be bicuspids and molars. As physicians, you will seldom have to extract anything forward of the bicuspids above, and not often forward of the molars below. You will be able generally, and you ought to seek to avoid especially, the extraction of all incisors and canines. Except in rare cases the dentist only should take this responsibility. The lower incisors seldom decay, and seldom need extraction by any one, and we must be exceeding slow to extract lower bicuspids, they being often of great importance for articulation with artificial teeth above. In selecting forceps, therefore, the weight of importance rests with those for molars generally, and upper bicuspids. What instruments are best and most generally available? What shall we buy? Negatively we are to make no investment and place no dependence in the molar forceps, styled "Universal." They are universally unfit for anything, but fracturing teeth at their necks. Whatever you may do without besides, have two pairs of upper molar forceps, right and left; and, in my judgment, there is nothing better than those known as the Harris', designed by the king among dentists, in his day. "Improvements" on his patterns have usually been injuries. Next, a pair of lower molar forceps, of either the Harris or Hutchinson pattern. The numbers of these instruments as manufactured by S. S. White, are: Uppers, 18; lower, Harris', 15; Hutchinson's, 47. Of these, I rather prefer the last named. And here let me say that, both for the sake of convenience, and because whatever instruments he sends out can be always relied on to be the best of their kind, I shall refer only to those manufactured by S. S. White by number. It is always safe to have an instrument manufactured by this the greatest manufacturer of instruments in the world; and his goods may be ordered directly from either of his four great depots, at Philadelphia, New York, Boston or Chicago.

For upper bicuspids have an instrument nearly straight, No. 11 or 40, the former preferably, and these will do for any ordinary case of extraction of upper canines or incisors. Here you are, with three pairs of forceps, supplied abundantly for all except unusual cases of extracting upper teeth, and one for all lower molars. Nos. 14 or 21 will complete the list for lower bicuspids, canines and incisors, in the very few cases where these have to be sacrificed by any one but the professional dentist.

Now, there ought to be, besides, a special instrument for upper wisdom teeth where these are small and overshadowed by the 2d molar, and the purchase of the Harris *dens sapientiæ* forceps, which is small enough to constitute a child's molar forceps, will be a good investment; or the sub-alveolar forceps, No. 77, recently invented by Dr. Tees, which is a valuable root forceps, and admirably adapted to difficult wisdom teeth extractions, will do equally as well.

For a general root forceps, Nos. 7 and 69 are about equally useful, and either will do for children's small teeth, and for lateral upper incisors, or crowded lower teeth of the second set.

Finally, and to complete the list, in order to meet numerous cases where it is well nigh impossible, or entirely so, to seize a root without nipping of a fragment of the alveolar process, or where there is a hollow crown so deeply decayed as to make fracture almost inevitable by the use of an ordinary instrument, add Parmly's bayonet shaped alveolar forceps, No. 32—applicable almost anywhere. With these eight instruments in possession, and thoroughly familiar with their qualities, you may consider the cases few and far between that you may not in some way successfully meet.

Of course there are many instruments that have their peculiar fitness for rare and difficult cases, but they are scarcely available for any but the specialist. A good instrument is like a good book in this, that there is nothing like being thoroughly familiar with it—better by far than a superficial and unfamiliar acquaintance with many.

Being instrumentally prepared for extracting, in cases where extracting cannot be avoided, the country physician should have certain other things, very essential to his own success and his patient's welfare, which he can obtain at small additional cost.

First of all, if he has not a physician's operating or invalid chair, he should have some sort of a head rest. This is a matter of necessity, not alone for operations upon teeth, but for examinations of the mouth and face, the nares, the eye, medical and and surgical applications to these, removal of foreign bodies, in short, anything to be done about the head and face. An excellent "rest," adjustable to any common chair, and to any position, can be bought of S. S. White, at a price varying according to style and finish from $7.50 to $15.00. It can be conveniently folded and carried into the country, and be often made useful in placing a patient in a position favorable for a critical examination, and accurate medication of the fauces in case of tonsillitis, diphtheria, etc. We all know by experience how nearly impossible it is to get a patient's head properly held by the unskilled assistant.

The next thing I shall mention, which no physician should be without, is a small non-magnifying mouth mirror. A very good and servicable one can be obtained for $1.00 or $1.50, and you may pay as much more as you wish for ornamental finish. If you have no laryngoscope you can make this answer some of the purposes of that instrument. You will find it very useful for throwing light into obscure corners of the mouth and throat, and affording views thereof, aside from its essential character as a dental mirror.

Next, pliers. If you are to examine and in any way treat teeth, you need a delicate pointed, nicely curved pair of these; and when you are once used to them you will find them available for many purposes beside; better by far for any delicate use than those generally found in surgical cases. This want may be well and cheaply supplied by purchasing the new "College" pliers for $1.00.

In order to make anything like an examination of a cavity of decay in a tooth, to determine its depth, direction, and bearing upon the life and salvation of the organ, you should have a few instruments adapted to opening into it and removing the debris. It will be necessary often to cut away the rough edge of enamel overhanging dead dentine, and for this purpose a good sharp chisel will be required. Among the many forms in use by dentists, there is one of more general availability, perhaps, than any other, represented in different sizes by Nos. 43, 44 and 45, of the S. S. W. set. Of these three instruments, No. 45 will answer most purposes, for the mere object of preparing the way for an examination. It can be used right and left, forward or backward, and can be sharpened on an oil stone till worn out. This will cost 60 or 70 cents. To remove the debris, a half dozen excavators and the same number of hand burrs (round) will be required, and of the former, Nos. 1, 5, 10, 18, 24 and 29, of the latter, 2, 4, 5, 7, 8 and 10 would be goood selections. Add to this list for opening into nerve canals, a half dozen small drills; and for inserting gutta percha stoppings, Nos. 1 and 4 S. S. W. burnishers. The whole cost of these will not exceed $5.

Additionally, for purposes of examination, you will need a syringe with a small curved point, for carrying a stream of water directly into cavities, to clean out the chips made by burrs and excavators. A hard rubber syringe good enough for the purpose, can be had for 75 cents.

For drying cavities, either to obtain a correct view for applying medicines or inserting temporary stoppings, it will be necessary to have on hand spunk or spongoid, and absorbent cotton. These articles cost 20 to 25 cts. per package, and their uses like many other things, in the way of dental apliances take a wide range. The physician and surgeon will find in ordinary practice nothing better than this "absorbent" cotton for making nice applications to tender and delicate surfaces, or for drying such parts preparatory to the application. It may be useful for washing or wiping out viscid and unwholesome secretions in cases of sore mouth and throat, in ridding the eyelids from the exudations of conjunctival inflammation, removing foreign substances from within the lid, and for cleansing the nares or external ear.

Although I have never so used it, it strikes me that either spunk, spongoid or the "absorbent" cotton, disinfected, would be excellent for absorbent dressings in the treatment of wounds. I believe the cotton, which is very coherent and readily formed into a firm string, would be excellent for draining punctured wounds. So much for the essential tools and appliances.

Now, a word as to a few medicaments and stoppings, adapted to giving present relief and temporary protection to an aching or sensitive tooth that may be saved. Every physician who treats teeth should have at hand the following:

Creasote and morphia..........................0.05 to 4.0
Clove oil " " ..........................0.05 to 4.0
Bottle of sandarac varnish.*
Carbolic acid; dissolved crystals.
A package of gutta percha pelletts.

I am strongly averse to advising any physician to have arsenic in readiness for use on teeth. It might, in rare cases, be used with advantage by skillful hands, but I am very sure that in the hands of any but a most competent and careful dentist, it is an agent far more likely to be potent for evil than good. You had better leave arsenic, as an applicant to teeth, in the hands of dentists only, and they had better use it but rarely. For my own part, whenever I find it necessary to extirpate a pulp, I much prefer giving an anæsthetic, and doing the operation quickly and at once with the dental engine, rather than use arsenic.

In this connection, I would remind any surgeon whose eye may follow this communication, of a fact he has probably seen mentioned before: that the dental engine, with its saws, bits, burrs and trephines, is capable of great usefulness in operations upon bone; in cases of necrosis, some resections, ununited fracture, overriding fragments, removal of tumors, etc., and any surgeon may find it occasionally of great advantage to call this instrument into requisition.

Dr. W. E. Garretson, of Philadelphia, has written recently a monograph specially upon this subject.

---

* This can be obtained ready prepared, or made by dissolving gum sandarac in alcohol, to a syrupy consistence.

Hints upon examination, extractions, alleviating treatment, and temporary stoppings, will occupy the space allotted to me for another paper.

(*To be concluded.*)

